# Influence of preventive dental treatment on mutans streptococci counts in patients undergoing head and neck radiotherapy

**DOI:** 10.1590/S1678-77572009000700003

**Published:** 2009

**Authors:** Lívia Buzati MECA, Fátima Regina Nunes de SOUZA, Helio Massaioshi TANIMOTO, Alvimar Lima de CASTRO, Elerson GAETTI-JARDIM

**Affiliations:** 1 Undergraduate student, School of Dentistry of Araçatuba, São Paulo State University-UNESP.; 2 Graduate student, School of Dentistry of Araçatuba, São Paulo State University-UNESP.; 3 DDS, MSc, Collaborator researcher, Department of Pathology and Clinic Propedeutics, School of Dentistry of Araçatuba, São Paulo State University-UNESP.; 4 DDS, MSc, PhD, Associate Professor, Department of Pathology and Clinic Propedeutics, School of Dentistry of Araçatuba, São Paulo State University-UNESP.

**Keywords:** Radiotherapy, Streptococcus mutans, Dental caries

## Abstract

The aim of this study was to evaluate the influence of chlorhexidine gluconate, sodium fluoride and sodium iodine on mutans streptococci counts in saliva of irradiated patients. Material and Methods: Forty-five patients were separated into three experimental groups and received chlorhexidine (0.12%), sodium fluoride (0.5%) or sodium iodine (2%), which were used daily during radiotherapy and for 6 months after the conclusion of the treatment. In addition, a fourth group, composed by 15 additional oncologic patients, who did not receive the mouthwash or initial dental treatment, constituted the control group. Clinical evaluations were performed in the first visit to dental clinic, after initial dental treatment, immediately before radiotherapy, after radiotherapy and 30, 60, 90 days and 6 months after the conclusion of radiotherapy. After clinical examinations, samples of saliva were inoculated on SB_20_ selective agar and incubated under anaerobiosis, at 37°C for 48 h. Total mutans streptococci counts were also evaluated by using real-time PCR, through TaqMan system, with specific primers and probes for *S. mutans* and *S. sobrinus*. Results: All preventive protocols were able to reduce significantly mutans streptococci counts, but chlorhexidine gluconate was the most effective, and induced a significant amelioration of radiotherapy side effects, such as mucositis and candidosis. Conclusion: These results highlights the importance of the initial dental treatment for patients who will be subjected to radiotherapy for head and neck cancer treatment.

## INTRODUCTION

Treatment of head and neck cancer (HNC) consists of surgery, radiotherapy (RT), and the association between them, besides the use of chemotherapy as an adjuvant in the treatment^14^. However, radiotherapy has been associated with several side effects, such as mucositis, changes in salivary gland function, radiation caries and especially osteoradionecrosis of the jaws^2^^,^^23^. These undesirable effects may affect treatment evolution and patient compliance with treatment. The occurrence of these reactions depends on the radiation dose, volume of irradiated tissue, fraction size, fractionation scheme, type of ionizing radiation, location of the irradiated area and other concomitant treatments^23^. In addition, individual aspects including age, systemic status, oral hygiene habits, tobacco and alcohol consumption^2^ also need to be considered.

The occurrence of radiation caries and mucositis is high as 40–100% of the irradiated patients^22^^,^^23^, producing extreme discomfort and compromising the acceptance^15^, continuity^21^ and intensification of RT^7^. Salivary gland dysfunction induced by RT results in hyposalivation, which may change the oral microbiota to a highly cariogenic microbiota, decrease clearance of carbohydrates from diet and organic acids produced by microorganisms, reduce buffering capacity, and impair remineralization of the tooth structure^9^^,^^18^.

In addition, patients who have xerostomia may consume a diet of soft, carbohydrate-rich foods, which may further increase the susceptibility to dental caries. Taken together, these changes may lead to rampant caries after RT^6^^,^^20^. In Brazil, three preventive schemes are followed by most of the radiotherapy centers for prevention of radiation caries and osteoradionecrosis: chlorhexidine gluconate (0.12%), sodium fluoride (0.5%, aqueous solution) and sodium iodine (2% in hydrogen peroxide 10 v/v). However, there are no microbiological evidences that these protocols are effective when associated to the oral hygiene, especially in a population composed mainly by people with low socioeconomic level.

Thus, the aim of this study was to evaluate the influence of these preventive protocols associated to the improvement of oral hygiene standards on mutans streptococci counts in 60 patients submitted to radiotherapy for treatment of head and neck cancer.

## MATERIAL AND METHODS

### Population

A total of 60 patients seen at the Department of Dentistry of the Barretos Cancer Hospital, SP, Brazil and the Megavoltage Radiotherapy Center, SP, Brazil, comprising 52 males and 8 females, aged 18–63 years (mean age 49.75 years), with histopathological diagnosis of malignant disease were included in this longitudinal study. Fifty patients presented squamous cell carcinoma, three with adenocarcinoma, six with Hodgkin lymphoma and one patient harbored liposarcoma.

All patients gave written informed consent to be recruited for this study, which was approved by the Research and Ethics Committee of the School of Dentistry of Araçatuba, São Paulo State University - UNESP (Proc. 136/2007). All patients had at least ten teeth after initial dental treatment (IDT) and were able to comply with the preventive clinical protocols. Patients with previous diagnosis of HIV infection, use of antibiotics 3 months before the first visit to the Cancer Hospital, uncontrolled significant cardiovascular, pulmonary, renal or hepatic disease were excluded.

Prior to radiotherapy, patients were separated randomly into four different groups:

Group I: patients were submitted to initial dental treatment (IDT), generally 3–4 weeks before RT, which consisted of extractions, restorations, scaling, and dental prophylaxis. These patients were instructed to use chlorhexidine gluconate (0.12%) once daily for additional oral biofilm control during RT and for 6 months after conclusion of the treatment. Oral hygiene instructions were reinforced at each visit;

Group II: after IDT, patients used sodium fluoride (0.5%, aqueous solution) daily and oral hygiene instructions were reinforced at each visit;

Group III: after IDT, the patients used sodium iodine (2% in hydrogen peroxide 10 v/v) once daily and oral hygiene instructions were reinforced at each visit;

Group IV: patients received no preventive dental treatment. It is important to highlight that they received no oral hygiene instructions before RT. Patients of this group were instructed by the oncologists to look for professional care in public dental clinics, but no one did it. They received medical treatment with no odontological assistance and received oral hygiene instructions only during and after RT.

The mean radiation dose received by the patients varied from 5.040 to 7.020 cGy, and the fractioning dose was 180 cGy. RT was carried out using a linear accelerator.

### Clinical procedures

In groups I, II or III, clinical examinations were performed at the first contact with the patient, before any dental treatment or oral hygiene instructions (stage 1), immediately after IDT (stage 2), before RT (stage 3), immediately after RT (stage 4), 30 days (stage 5), 60 days (stage 6), 90 days after RT (stage 7) and 6 months after RT (stage 8). The oral hygiene status was assessed using the plaque index (PI)^19^. In group IV, clinical examinations were performed just before RT, 3 weeks after the beginning of RT, immediately after, 30 days and 6 months after RT.

### Collection of clinical samples, microbial isolation and enumeration

Whole resting saliva was collected from each patient before IDT, immediately after IDT, before RT, immediately after RT, 30, 60, 90 days and 6 months after RT, for mutans streptococci enumeration. Saliva was collected through the draining method; patients were placed in a quiet room, asked not to drink, eat or clean their mouths 1 h before saliva collection and instructed not to swallow any saliva during the collection period^3^. Test tubes containing the samples were immediately placed in a refrigerator (for culture) or liquid nitrogen (real-time PCR).

Laboratory processing was performed within 2 h. After mechanical mixing, samples were serially diluted and plated on selective SB_20_ agar, incubated anaerobically (90% N_2_ + 10% CO_2_), for 48 hours, at 37°C, for mutans streptococci enumeration. Bacterial identification was carried out by means of bacteria and colony morphology analyses as well as biochemical tests. After evaluation of mutans streptococci counts by culture, the caries risk of each subject was determined^24^.

### Quantification of mutans streptococci by real-time PCR

The presence and quantification of mutans streptococci were also confirmed by real-time PCR. After extraction of bacterial DNA from saliva by QIAamp DNA Mini Kit (Qiagen, Duisburg, Germany), real-time PCR was carried out using a Rotor Gene 6000 (Corbett Life Science, Mort Lake, New South Wales, Australia). Each PCR was performed in duplicate using a total volume of 25 μl, containing 12.5 μl 2X Taqman Universal Master Mix (Applied Biosystems), 0.2 μl each of forward and reverse primers (final concentration, 200 nM each), an appropriate concentration of Taqman probe (final concentration 100 nM), 2 μl of template DNA solution and an appropriate volume of sterilized DNase-RNase-free water. Amplification reactions were performed by an initial denaturation at 94°C for 10 min, 40 cycles at 95°C for 15 s and 60°C for 1 min. Primers were designed according to those described in literature for *S. mutans* and *S. sobrinus*^25^.

### Statistical analysis

Statistical analysis was performed using the software Statistical Package for the Social Sciences (SPSS Inc., v.13, Chicago, IL, USA). Quantitative variables (mean and standard deviation) were analyzed with Student’s t-test. Multiple comparisons were carried out by means of Kruskall-Wallis test, while dichotomous variables were analyzed by Mann-Whitney, Chi-square or Fisher’s exact tests. Difference of P < 0.05 was considered statistically significant.

## RESULTS

Unfortunately, out of the 60 patients initially examined, 10 did not conclude RT and 11 other patients were not in physical conditions to be submitted to final intra-oral examinations. Oral manifestations associated to radiotherapy are presented in Table 1. Before RT, oral mucositis and dermatitis were not observed. Erythematous candidosis was detected in one patient of group IV and xerostomia was reported by two patients (group II and group IV). After RT, mucositis, xerostomia, and dermatitis were widely disseminated, irrespective of the experimental group, and except for candidosis, there were no statistically significant differences between groups regarding these side effects of RT.

**Table 1:** Oral status of the experimental groups at different periods of analysis

Clinical Condition		Occurrence of radiotherapy side effects N (%)			
		Before RT^1^	After RT^2^	30 d after RT^3^	6 months after RT^4^
**Mucositis**					
	Group I	0 (0.0)	12 (92.31)	8 (61.54)	4 (40.0)
	Group II	0 (0.0)	10 (90.91)	7 (63.63)	3 (33.33)
	Group III	0 (0.0)	11 (78.57)	8 (66.67)	4 (36.36)
	Group IV	0 (0.0)	10 (83.33)	8 (72.73)	7 (77.78)
**Dermatitis**					
	Group I	0 (0.0)	12 (92.31)	8 (61.54)	1 (10.0)
	Group II	0 (0.0)	11 (100.0)	8 (88.89)	2 (22.22)
	Group III	0 (0.0)	11 (78.57)	7 (63.64)	0 (0.0)
	Group IV	1 (6.67)	12 (100.0)	7 (77.78)	2 (22.22)
**Candidosis**					
	Group I	0 (0.0)	3 (23.08)	1 (7.69)	0 (0.0)
	Group II	0 (0.0)	3 (27.27)	2 (18.18)	1 (9.09)
	Group III	0 (0.0)	4 (28.57)	1 (8.33)	0 (0.0)
	Group IV	1 (6.67)	8 (66.67)	5 (45.45)	3 (33.33)
**Xerostomia**					
	Group I	0 (0.0)	12 (92.31)	10 (76.92)	8 (80.0)
	Group II	1 (6.67)	11 (100.0)	10 (90.91)	6 (75.0)
	Group III	0 (0.0)	12 (85.71)	9 (75.0)	7 (63.63)
	Group IV	1 (6.67)	11 (91.67)	8 (72.73)	7 (77.78)

1 Number of patients in the different groups just before radiotherapy: Group I, N= 15; Group II, N= 15; Group III, N= 15; Group IV, N= 15. Total = 60.

2 Number of patients in the different groups immediately after radiotherapy: Group I, N= 13; Group II, N= 11; Group III, N= 14; Group IV, N= 12. Total = 50.

3 Number of patients in the different groups 30 days after conclusion of radiotherapy: Group I, N= 13; Group II, N= 11; Group III, N= 12; Group IV, N= 11. Total= 47.

4 Number of patients in the different groups 6 months after conclusion of radiotherapy: Group I, N= 10; Group II, N= 9; Group III, N= 11; Group IV, N= 9. Total= 39.

In Group IV, immediately after RT, candidosis was diagnosed both in its pseudomembranous (two cases) and erythematous variants (six cases), while patients of the other groups presented only chronic erythematous candidosis. The occurrence of candidosis was significantly higher in group IV (*P*= 0.031).

The incidence of RT side effects was reduced 30 d after conclusion of the treatment, but occurrence of candidosis remained higher in group IV than in other groups. Six months after RT conclusion, xerostomia and mucositis constituted the most common alterations in the oral cavity, although their severity evidenced a mild reduction in most patients. In group IV, the reduction of mucositis and candidosis was slower than reported in other groups, and 77.78% of the patients presented this condition 6 months after RT. Moreover, in group IV, mucositis was statistically associated with xerostomia (*P* < 0.001), while in the other groups xerostomia was restricted to approximately 30–40% of the patients, 6 months after RT.

Xerostomia, dermatitis and mucositis were not prevented by any particular protocol, and all groups evidenced similar results (Table 1), while all preventive protocols reduced the occurrence of candidosis. It was also observed that in patients presenting clinical signs of mucositis 6 month after RT conclusion, mucositis level I and II predominated; while immediately after RT, most patients harbored mucositis level II and, especially, level III.

Initial plaque index values were very high in groups I, II, and III, ranging from 3.02 ± 0.54 (group I) to 3.46 ± 0.98 (group II), with no significant differences between groups. However, there was a gradual reduction of these values over the clinical follow-up period and no significant differences were observed between groups I, II and III, while group IV showed 3.25 ± 0.84 before RT and 2.86 ± 0.61, six months after RT treatment. Statistical analysis of the plaque index values revealed a significant reduction of dental plaque in groups I, II, and III (*P*= 0.0026), but in group IV the reduction was not statistically significant (*P*= 0.232). The improvement of oral hygiene standards was more pronounced just before and after RT, reinforcing the need for continuous follow-up.

In relation to mutans streptococci counts, they were very high at baseline, but initial dental treatment as well as all preventive protocols used during the study were able to reduce acidogenic cocci. The mutans streptococci counts are shown in Table 2. No statistical difference between values in each group was observed before RT, but the data 30 d after RT revealed a significant reduction of streptococci in group I (chlorhexidine) in comparison with group II (*P*= 0.03), group III (*P*=0.001) or group IV (*P*< 0.001). After the beginning of RT, group IV evidenced significantly higher counts of cariogenic cocci in relation to the other groups (P<0.001), evidencing the role of initial dental treatment in reducing cariogenic cocci. Comparison between groups II and III showed statistically non-significant differences (*P*= 0.9952).

**Table 2:** Mean counts of mutans streptococci in the experimental groups during the study

Groups	Mean mutans streptococci counts x10^5^ ± Standard deviations x10^5^				
	Baseline^1^	before RT	after RT	30 d after RT	6 months after RT
**Group I**					
Culture^2^	8.7 ± 4.6	1.9 ± 3.7	2.3 ± 1.8	2.0 ± 0.56	7.5 ± 3.1
Real-time PCR^3^	11.4 ± 5.4	2.1 ± 4.8	2.9 ± 1.9	2.6 ± 0.7	9.2 ± 3.1
**Group II**						
Culture	16 ± 4.9	4.4 ± 3.7	5.7 ± 1.8	5.2 ± 2.9	9.8 ± 5.1
Real-time PCR	23 ± 12.5	5.1 ± 4.7	7.0 ± 5.6	7.9 ± 6.3	14 ± 11.3
**Group III**					
Culture	21 ± 9.2	12 ± 3.7	5.4 ± 5.1	15.8 ± 6.2	16.2 ± 3.8
Real-time PCR	37 ± 12	9 ± 8.3	6.7 ± 4.9	15.0 ± 3.1	18.8 ± 7.1
**Group IV**					
Culture	—4	8.5 ± 4.8	18.7 ± 9.6	81 ± 41	77 ± 22.4
Real-time PCR	—	7.8 ± 5.2	15.3 ± 13.4	123 ± 45.2	83 ± 24.5

1 Immediately before dental treatment.

2 CFU/mL.

3 DNA copies/mL. Total *S. mutans* + *S. sobrinus*.

4 Group IV: patients did not receive initial dental treatment.

At baseline, most patients of groups I, II and III were at high-risk for dental caries or presented microbial overgrowth on agar plates. There was a slight caries risk reduction after IDT and this phenomenon was sustained during RT, except for group IV (Table 3).

**Table 3:** Effects of preventive protocols on dental caries risk in irradiated patients. Results of mutans streptococci counts were obtained by culture

Group	Caries risk			
	1low risk N(%)	moderate risk N(%)	high risk N(%)	plate overgrowth N(%)
**Group I**				
Before IDT	0 (0.0)	2 (13.33)	7 (46.67)	6 (40.0)
Before RT	1 (6.67)	3 (20.0)	9 (60.0)	2 (13.33)
After RT	0 (0.0)	4 (30.77)	9 (69.23)	0 (0.0)
6 months after RT	0 (0.0)	3 (30.0)	4 (40.0)	3 (30.0)
**Group II**				
Before IDT	0 (0.0)	0 (0.0)	8 (53.33)	7 (46.67)
Before RT	2 (13.33)	3 (20.0)	9 (60.0)	1 (6.67)
After RT	0 (0.0)	3 (27.27)	8 (72.72)	0 (0.0)
6 months after RT	0 (0.0)	2 (22.22)	4 (44.44)	3 (33.33)
**Group III**				
Before IDT	0 (0.0)	1 (6.67)	8 (53.33)	6 (40.0)
Before RT	1 (6.67)	2 (13.33)	8 (53.33)	4 (26.67)
After RT	0 (0.0)	0 (0.0)	10 (71.43)	4 (28.57)
6 months after RT	0 (0.0)	2 (18.18)	5 (45.45)	4 (36.36)
**Group IV**				
Before IDT	—a	—	—	—
Before RT	0 (0.0)	2 (.0)	8 (53.33)	5 (33.33)
After RT	0 (0.0)	0 (0.0)	5 (41.67)	7 (41.67)
6 months after RT	0 (0.0)	0 (0.0)	3 (33.33)	6 (66.67)

1 Low risk, mutans streptococci counts <10^4^ CFU/mL of saliva; moderate risk, mutans streptococci counts 10^4^ - <10^5^ CFU/ mL of saliva; high risk, mutans streptococci counts 10^5^ - <10^6^ CFU/mL of saliva; plaque overgrowth, mutans streptococci counts =10^6^ CFU/mL of saliva;

a Group IV: patients did not receive initial dental treatment.

## DISCUSSION

Dental caries risk is a serious problem for patients undergoing RT for head and neck cancer^9^^,^^14^^,^^23^. Carious lesions develop rapidly, and advanced destruction of the tooth structure can be observed as fast as several weeks or months after RT. Therefore, preventive measures before, during, and after RT are necessary and should include instructions regarding a noncariogenic diet, regular oral hygiene, and application of chemical compounds to prevent microbial accumulation in the biofilm or mineral loss of the dental structures^9^. However, literature^8^^,^^10^ has shown that caries and cariogenic microbiota can be controlled by topical fluorides and chlorhexidine application. In the present investigation, chlorhexidine produced the most noticeable changes in the cariogenic microbiota of the patients.

In the present study, most patients with head and neck cancer are middle aged adult males who were chronic tobacco and alcohol consumers and had advanced tumors located especially in the floor of the mouth and tongue. In these patients, dental treatment before RT is necessary to avoid dental extractions and prevent osteoradionecrosis and other traumatic sequelae during and after RT^2^^,^^16^. This is particularly true for patients with low socioeconomic conditions who show poor oral hygiene status^2^.

At baseline, the population evaluated in this study presented an initial very high risk for dental caries and although a significant reduction in levels of mutans streptococci was achieved especially in group I patients, the occurrence of new lesions of dental caries was observed in some patients of all groups, but especially in group IV.

Patients with high-risk for dental caries who have medium to high levels of mutans streptococci should use an antibacterial mouthrinse^11^. Currently, the most effective antibacterial mouthrinse against cariogenic bacteria is chlorhexidine. High-risk adults should rinse daily with 10 mL for 1 minute at bedtime for 1 week. This should be done for 1 week every month for up to 6 months. If used only 1 week per month, staining of the teeth and oral mucosa should be a minimal issue. Compliance is also a major issue with this product, which is why it should only be used for 1 week per month^11^. However, in the present study, all patients showed high risk for caries at baseline and this risk would obviously increase by irradiation, then the use of chlorhexidine was recommended during all the experiment.

The slight reduction in the levels of mutans streptococci observed in patients using sodium fluoride (group II) may have occurred not by the direct antimicrobial activity of the chemical agent, but due to the inhibitory activity which it carries on the enzymes related to saccharolytic metabolism. This could represent an ecological disadvantage for acidogenic cocci, as many oral microorganisms adhere better to acidic pH. Thus, the reduction of carbohydrate fermentation may have contributed to the slight reduction of mutans streptococci in the biofilm and mucositis incidence, avoiding acidification of the oral environment.

The use of topical fluoride to reduce caries has become standard practice in RT patients^9^, but as all preventive protocols, this protocol is very sensitive to patients’ compliance^9^. It has been estimated that patients must follow an application frequency of at least 70% to prevent decay^13^. However, compliance with fluoride application in carriers by the population with head and neck cancer is generally thought to be poor^4^^,^^13^^,^^18^ and this phenomenon may be due to the inconvenient method of application. Although fluoride gel can provide additional benefits, as it maintains fluoride in the oral cavity for a longer period, our experience showed that topical aqueous fluoride solution induces a higher compliance, thus this protocol was chosen.

Other compounds used until recently in the prevention of radiation caries and osteoradionecrosis, such as sodium iodide prepared in hydrogen peroxide have been discouraged due to their suspected carcinogenicity and toxicity on fibroblasts, delaying the repair process^5^.

Many head and neck cancer patients have poor oral hygiene^2^^,^^17^ and patient adherence to the preventive protocols is closely correlated with follow-up visits^6^^,^^9^. Therefore, patient care must be individualized with evaluation at regular intervals to determine the caries risk and evolution, in order to preserve adequate oral health status^9^. In this study, patients were instructed to maintain a monthly visit routine to the dental office and this regimen probably interfered with patients’ compliance, thus improving significantly the clinical outcome of the preventive protocols.

After RT, no single case of osteoradionecrosis was observed, probably due the time span between teeth extractions and the beginning of RT^23^ in groups I, II and III, since wound healing during RT represents a high risk for the onset of osteoradionecrosis^1^^,^^12^. In group IV, due to lack of time for completion of the dental treatment, dental extractions were postponed indefinitely.

The occurrence of new caries in the experimental groups was much lower than initially expected and, in spite of the fact that no statistically significant findings were identified between caries experience and history of fluoride, iodine or chlorhexidine use, all preventive protocols were considered effective in the prevention of radiation caries and osteoradionecrosis.

## CONCLUSIONS

Chlorhexidine was the most efficient mouthrinse to reduce mutans streptococci in the saliva of head and neck cancer patients undergoing radiotherapy treatment; it also ameliorated oral mucositis and eliminated oral candidosis in the experimental groups. The results evidenced the great importance of the dental team and initial dental treatment as a measure to reduce the severity and extension of radiotherapy side effects in the oral cavity.
